# Investigating the Association Between Heatstroke Mortality and Climatic Factors in Bangladesh: An Ecological Time Series Study

**DOI:** 10.1002/hsr2.72510

**Published:** 2026-05-13

**Authors:** Md. Mamun Miah, Farjana Haque Pingki, Sarmin Akhter, Mehedi Hasan, Minhaz Uddin, Rakib Rahman, Tanzir Ahamed Raihan, Md. Shiblur Rahaman

**Affiliations:** ^1^ Department of Statistics Noakhali Science and Technology University Noakhali Bangladesh; ^2^ Research and Training Division, Bangladesh Institute for Research and Statistical Training Mirpur Dhaka Bangladesh; ^3^ Department of Fisheries and Marine Science Noakhali Science and Technology University Noakhali Bangladesh; ^4^ Department of Environmental Science and Disaster Management Noakhali Science and Technology University Noakhali Bangladesh

**Keywords:** Bangladesh, heatstroke mortality, humidity, negative binomial regression, temperature

## Abstract

**Background and Aims:**

Heatstroke is a life‐threatening illness with increasing global prevalence, largely influenced by weather conditions. It is a major health concern in Bangladesh, where the climate is especially hot and humid during the summer months. Temperature and humidity are two key factors of heat‐related health outcomes. This study aims to analyze the effects of these climatic factors on heatstroke mortality in Bangladesh.

**Methods:**

The study utilized secondary data collected from April 1, 2024, to May 31, 2024. Daily heatstroke mortality cases and corresponding climatic factors were analyzed. Descriptive statistics were used to summarize the data, while Kendall's tau *b* and Spearman's *ρ* were applied to measure the bivariate associations. The Negative Binomial (NB) regression model was employed to evaluate the impact of daily maximum temperature and relative humidity on heatstroke mortality.

**Results:**

The findings revealed a positive association between climatic factors and heatstroke mortality. Maximum temperature was associated with higher risk of mortality (IRR: 1.71, 95% CI: 1.34–2.17), as was relative humidity (IRR: 1.05, 95% CI: 1.01–1.08). Specifically, a one‐unit increase in the daily maximum temperature and relative humidity was associated with a 71% and 5% increase in the rate of heatstroke mortality, respectively.

**Conclusion:**

Climatic factors, mainly temperature and humidity, play a significant role in heatstroke mortality in Bangladesh. These findings highlight the need for targeted public health interventions and climate change policies to reduce heat‐related health risks, especially among vulnerable populations.

## 1 Introduction

Heatstroke is the most severe form of heat‐related illness and represents a life‐threatening condition resulting from the body's inability to regulate its core temperature [1]. It typically occurs after prolonged exposure to high environmental temperatures, leading to critical damage to organs and, in severe cases, death. In recent decades, heatstroke has become a severe global public health issue, largely due to the increasing number and intensity of heatwaves.

A heat wave is defined by the World Meteorological Organization (WMO) as five or more days of sustained heat with a daily maximum temperature that is five degrees Celsius (5°C) greater than the usual maximum temperature, often coupled with elevated humidity levels [2]. In some regions, temperatures exceeding 45°C for two consecutive days, are also considered heatwave [3]. Climate change is the crime factor of rising global temperatures and has potentially increased the frequency, duration, and severity of heatwaves [4].

A growing number of literatures indicate that heatwaves are linked with high risk of injury and death. Extreme heat directly or indirectly affects human health, leading to increased morbidity and mortality [5, 6]. Historical heatwave events have resulted in substantial loss of life worldwide, highlighting the severity of this issue [7]. Furthermore, temperature related mortality rates vary by location and population characteristics [8]. Several studies investigated the association between temperature and mortality across different regions [9, 10].

In addition to environmental conditions, individual and socioeconomic characteristics also influence vulnerability to heat‐related illnesses such as heatstroke. Factors such as age, sex, occupation, and socioeconomic status can affect both exposure to extreme heat and the ability to cope with heat stress. Older adults, particularly those aged 70 years and above, have been reported to experience higher rates of heat‐related emergency transport [11]. Demographic characteristics, including the male‐to‐female ratio, together with environmental factors such as solar radiation, have also been identified as key predictors of heatstroke outbreaks in urban areas [12]. Moreover, socioeconomic disparities can also limit people's ability to adapt to heat. Future projections indicate that heatstroke cases will increase significantly in the future, with age and sex interacting with environmental conditions to influence risk patterns [13].

Bangladesh, located in South Asia, is particularly vulnerable to the impacts of heatwaves due to its tropical monsoon climate. The country frequently experiences high temperatures and humidity, especially during the summer months from March to June of the year, creating an environment conducive to heat‐related illnesses. Historical data indicate that Bangladesh has experienced extreme temperature events, including recorded maximum temperatures of 45.1°C in the month of May, which is considered as the highest in recent decades [14]. Heat‐related mortality is becoming a rising concern in the country; however, comprehensive studies examining the relationship between climatic variables, and heatstroke mortality remain limited.

Despite the increasing health risks caused by heatwaves, there is a lack of focused research investigating the combined effects of heatwaves, temperature, and humidity on heatstroke mortality in Bangladesh. This study aims to address this gap by investigating the association between heatwaves, maximum temperature, relative humidity, and heatstroke mortality in Bangladesh. To the best of our knowledge, this is the first study in the country to systematically evaluate the influence of climatic factors on heatstroke‐related deaths. The findings of this research are expected to provide valuable evidence to inform public health policies and intervention strategies aimed at reducing heat‐related health risks, especially among vulnerable populations. As global temperatures continue to rise, understanding the interplay between climatic and socioeconomic factors is essential for enhancing resilience and reducing the burden of heat‐related illnesses.

## 2 Materials and Methods

### 2.1 Study Area

Bangladesh stretches from 20° 34′ N to 26° 38′ N latitude and 88° 01′ E to 92° 41′ E longitude and is the world's largest deltaic and flat topographical country. It has a humid monsoon climate with distinct seasonal variations in temperature and rainfall [15], and monsoon regions in South Asia commonly experience humid heatwaves linked to seasonal rainfall cycles [16].

The country undergoes four main seasons: pre‐monsoon (March to May), monsoon (June to September), post‐monsoon (October to November), and winter (December to February) [17]. The pre‐monsoon summer season is the most intense, with temperatures often rising above 42°C [18]. According to the Bangladesh Meteorological Department (BMD, 2020), the long‐term average minimum and maximum temperatures over the country are 21°C and 29°C, respectively. April is typically proposed as the warmest month of the season; the average maximum temperatures are 27°C to 31°C.

During the pre‐monsoon period, humidity levels are relatively lower compared to the monsoon season, sometimes dropping to around 57%, whereas monsoon humidity often exceeds 80%. Wind speeds during this season typically range from 8 to 16 km/h [19].

### 2.2 Data Source

Mortality data were obtained from two sources: (i) aggregate reports from the Directorate General of Health Services (DGHS), which provided total counts of heatstroke‐related deaths over specific time periods, and (ii) media reports, which gave more detailed district‐level or daily figures.

Climatic data, including daily maximum temperature (°C) and relative humidity (%), measured at 2 m above ground level, were obtained from the NASA POWER Data Access Viewer (https://power.larc.nasa.gov/).

### 2.3 Construction of Daily Heatstroke Mortality Data

Daily heatstroke mortality data for the study period (April 1 to May 31, 2024) were obtained from multiple sources with varying levels of temporal resolution.

For April 2024, data were directly available from publicly accessible reports and media sources, and these were used without modification.

For May 2024, however, only aggregated mortality counts were available from the Directorate General of Health Services (DGHS), reported over specified time intervals rather than as daily observations. Since the statistical modeling framework used in this study requires daily data, it was necessary to construct daily mortality counts for this period.

To achieve this, DGHS cumulative counts were treated as anchor totals. Daily counts within these periods were allocated as follows: (i) when no temporal detail was available, deaths were distributed uniformly across the period; (ii) when media reports indicated clustering on specific dates, reported counts were assigned to those dates and remaining counts were distributed across adjacent days; and (iii) single‐day reported deaths were incorporated directly.

**Figure 1:**
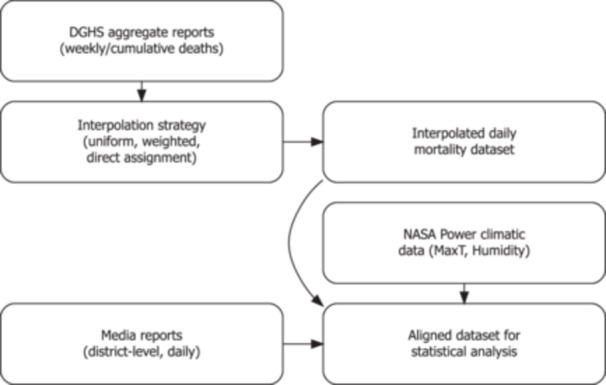
Data processing flow diagram.

This approach ensured consistency with official totals while improving temporal resolution. The data processing workflow is illustrated in Figure 1.

### 2.4 Statistical Analysis

The primary outcome variable was the daily number of heatstroke‐related deaths, which was a count variable. Descriptive statistics, including mean, standard deviation (SD), minimum, and maximum, were calculated for all study variables.

The distribution of the outcome and predictors was assessed using the Shapiro–Wilk normality test, which indicated non‐normality (p<0.001). Therefore, Kendall's tau_*b* and Spearman's *ρ* correlation coefficients were applied to evaluate the bivariate relationship between the heatstroke mortality and climatic variables. These analyses were conducted for exploratory purposes and to provide preliminary insight into the direction and strength of associations prior to multivariable modeling.

To assess the multivariable effects of climatic factors on heatstroke mortality, generalized linear models (GLMs) were employed. A poisson regression model was initially fitted, which showed overdispersion (variance exceeding the mean and dispersion statistics > 1). Therefore, alternative count models, including Negative Binomial (NB), Zero‐Inflated Negative Binomial (ZINB), and the Zero‐Inflated Poisson (ZIP) were considered. Zero‐inflated models were considered to account for potential excess zeros in daily mortality counts. Model selection was primarily based on the Bayesian Information Criterion (BIC), where lower values indicate better model fit.

Moreover, to assess whether zero‐inflated models provide statistically significant improvement in model fit compared to standard count models, Vuong's test was applied. The results did not show any significant advantage of zero‐inflated models over the standard count models.

Based on BIC and supporting diagnostic measures, the Negative Binomial (NB) model was selected as the final model.

**Figure 2:**
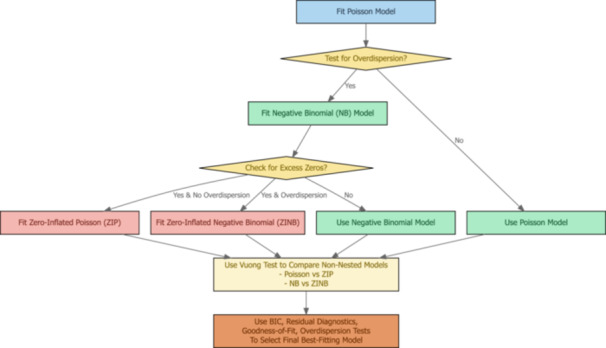
Flow diagram of model selection for count regression analysis.

Model adequacy was assessed using residual diagnostics and goodness‐of‐fit measures. Results are reported as incidence rate ratios (IRRs) with 95% confidence intervals (CIs). All statistical tests were two‐sided, and statistical significance was defined at *α* = 0.05. All analyses were conducted using R statistical software (version 4.1.2). The model selection procedure is presented in Figure 2.

## 3 Results

### 3.1 Descriptive Analysis

Descriptive statistics for daily heatstroke mortality and meteorological variables during April 1 to May 31, 2024 are shown in Table 1. The average number of daily deaths was 1.21 (SD = 1.74), with a median of 1.0. Mortality counts were generally low, although occasional peaks were observed a maximum of 10 deaths. The interquartile range (0–1) indicates that most days experienced zero to one death.

**Table 1:** Descriptive statistics of daily heatstroke mortality and climatic factors for April 01, 2024, to May 31, 2024.

Variables	Number of deaths	Maximum temperature	Relative humidity
Mean (SD)	1.213 (1.74)	30.40 (1.59)	69.13 (11.72)
Median	1.0	30.11	69.61
1Q	0.0	29.46	60.73
3Q	1.0	31.69	76.37
Lowest	0	26.57	44.51
Highest	10	33.49	90.36

The mean daily maximum temperature was 30.40°C (SD = 1.59), with values ranges from 26.57°C to 33.49°C. The interquartile range (29.46°C to 31.69°C) suggests relatively stable temperature conditions during the study period. Relative humidity showed greater variability, with a mean of 69.13% (SD = 11.72) and a range from 44.51% to 90.36%. The interquartile range (60.73% to 76.37%) indicates moderate to high humidity levels on most days.

**Figure 3:**
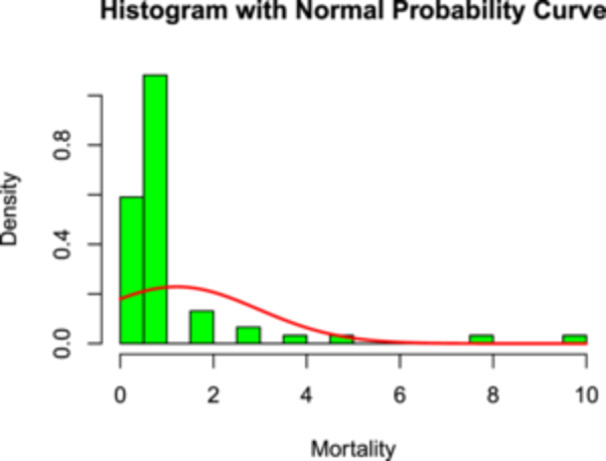
Histogram and density plot of heatstroke mortality.

The distribution of daily heatstroke mortality (Figure 3) was right‐skewed, with most observations concentrated at lower values and a small number of high mortality days. The Shapiro–Wilk normality test confirmed that the mortality data were not normally distributed (p<0.001).

### 3.2 Bivariate Analysis

The correlation between heatstroke mortality and environmental factors is presented in Table 2. Both maximum temperature and relative humidity showed positive associations with mortality. Spearman's *ρ* was 0.33 and 0.15 for maximum temperature and humidity respectively, indicating a stronger relationship with temperature.

**Table 2:** Correlation of heatstroke mortality with selected meteorological variables.

Meteorological variables	Heatstroke mortality	
	Kendall's_Tau_*b*	Spearman's *ρ*
	Corr. coef. (*r*)	Corr. coef. (*r*)
Relative humidity (%)	0.107^a^	0.151^a^
Maximum temperature (°C)	0.241^a^	0.331^a^

^a^ Indicates a significant correlation at a 0.01 level.

### 3.3 Effects of Meteorological Factors on Heatstroke Mortality

Based on the Bayesian Information Criterion (BIC) values (Table 4), the Negative Binomial (NB) regression model provided the most parsimonious model with the best balance between model fit and complexity. Therefore, NB model was selected for further analysis.

The regression results are presented in Table 3. Maximum temperature and relative humidity were both significantly associated with heatstroke mortality. A 1°C increase in maximum temperature was linked with a 71% increase in expected mortality (IRR: 1.71, 95% CI: 1.34–2.17, *p* < 0.001) (Table 5). similarly, a 1% increase in relative humidity was associated with a 5% increase in expected mortality (IRR: 1.05, 95% CI: 1.01–1.08, *p* < 0.001) (Table 5).

**Table 3:** Summary statistics of the estimated Negative Binomial (NB) regression model.

Predictors	Estimate	Std. Error	*z* value	p‐value
(Intercept)	−19.54	4.76	−4.10	<0.001
Maximum temperature (°C)	0.53	0.12	4.34	<0.001
Relative humidity (%)	0.05	0.01	2.61	<0.001

**Table 4:** Goodness of fit statistics for selecting the best‐fitted model.

Model	BIC
Poisson	180.22
Zero‐inflated poisson	182.60
Negative binomial	179.27
Zero‐inflated negative binomial	182.99

**Table 5:** Incidence rate ratio of each independent variable in the negative binomial regression model.

	Incidence rate ratio (IRR)	95% CI of IRR		
		Lower limit	Upper limit	*p*‐ value
(Intercept)	3.27 × 10^−9^	2.91 × 10^−13^	3.68 × 10^−5^	<0.001
Maximum temperature (°C)	1.71	1.34	2.17	<0.001
Relative humidity (%)	1.05	1.01	1.08	<0.001

### 3.4 Model Diagnostics and Residual Analysis

**Figure 4:**
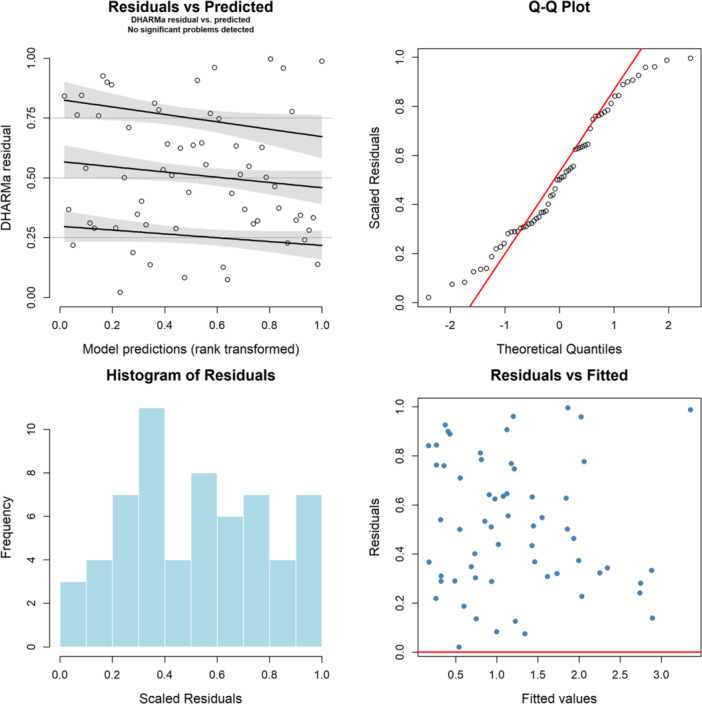
Diagnostic plots for the negative binomial regression model.

Model diagnostics for the negative binomial model (Figure 4) indicated a good fit. There was no multicollinearity evidenced from Variance Inflation Factor (VIF) values (all values<2). No overdispersion observed by a dispersion parameter of 1.0004 and a deviance‐to‐residual degrees‐of‐freedom ratio of 0.888. Simulated residuals generated using R package “DHARMa” confirmed model adequacy, where statistical tests revealed no overdispersion (p=0.264), no significant zero‐inflation (p=0.064), and no outliers (p=1). Furthermore, graphical inspection of residual diagnostics supported these findings. Residuals vs. predicted and fitted values centered around zero, Q–Q plots showed approximate uniformity, and histograms displaying well‐behaved distributions. Collectively, these results indicate that the negative binomial model provides a robust and appropriate representation of the data.

## 4 Discussion

This study investigated the association between climatic factors and heatstroke mortality in Bangladesh. The results indicate that both maximum temperature and relative humidity are positively associated with daily heatstroke mortality, focusing their importance in understanding heat related health risks in tropical settings.

The results from the Negative Binomial model show that higher temperatures are significantly linked with increased heatstroke mortality. Specifically, each 1°C increase in maximum temperature was associated with an increase in the expected mortality rate (IRR = 1.71). This outcome is consistent with previous studies demonstrating that elevated extreme temperatures and heatwaves substantially increase mortality risk, particularly during extreme heat events [20]. Similar positive associations between high temperatures and daily mortality have been observed across diverse geographic regions [5]. Systematic reviews further confirm that heatwaves significantly increase both mortality and morbidity, especially among vulnerable populations [21]. In South Asia, mortality has been shown to rise sharply beyond specific temperature threshold levels [22].

Supporting evidence from Kanto region of Japan depicts that daily maximum temperatures exceeding 35°C are strongly associated with increased mortality [23]. Another study report exponential increases in mortality when temperatures exceed 38°C [24]. Moreover, heat associated mortality is influenced not only by extreme daily temperatures but also by long term climatic condition. Populations in cooler regions may experience higher mortality at relatively lower temperatures due to lower acclimatization [25]. Time‐stratified case‐crossover studies further revealed that both minimum and maximum daily temperatures are associated with increased heatstroke incidence, especially for non‐exertional cases [26].

In addition to temperature, relative humidity was identified as a significant predictor of heatstroke mortality. The estimated incidence rate ratio (IRR = 1.05) indicates that a 1% increase in relative humidity is associated with a 5% increase in the expected rate of heatstroke mortality, holding other factors constant. This finding highlights the physiological impact of humidity, which impairs the body's ability to dissipate heat through evaporation. Previous studies have demonstrated that combined exposure to high temperature and humidity significantly increases heat stress and mortality risk [27]. For instance, a humidex values above 30°C have been associated with increased health risk, while levels above 40°C may lead to severe discomfort and adverse health outcomes [28]. Additionally, evidence suggests that mortality tends to be higher on days with elevated humidity for a given maximum temperature, underscoring the interaction between these climatic factors [29]. Elevated relative humidity has also been identified as a key contributor to thermal discomfort and heat related health risks [30].

From a public health perspective, these findings underscore the importance of jointly considering temperature and humidity in the development of heat‐health warning systems and intervention strategies. In Bangladesh, where high temperature and humidity frequently coexist during the pre‐monsoon season, targeted approaches such as early warning systems, public awareness campaigns, and adaptive measures are essential to mitigate heat‐related health risks.

### 4.1 Limitation of the Study

There are several limitations to this study that should be considered when interpreting the findings. First, the analysis was based on a relatively short study period (April–May 2024) and restricted to the pre‐monsoon season. This limited temporal coverage may affect the generalizability and stability of the estimated associations, as heatstroke events may also occur in other seasons. Future studies using longer time‐series data across multiple seasons and years would provide more robust estimates.

Second, the daily heatstroke mortality data were obtained from sources with varying temporal resolution. While daily data were directly available for April 2024, the data for May 2024 were partially derived from aggregated reports by distributing counts across individual days. This data construction approach may introduce measurement uncertainty and could influence model estimates, particularly for the latter period. Therefore, the findings should be interpreted with caution, and future research should prioritize the use of consistently recorded daily mortality data.

Third, the study focused primarily on climatic variables, specifically maximum temperature and relative humidity, and did not incorporate other relevant environmental and non‐environmental factors. Important potential confounders such as air pollution, age, sex, socioeconomic status, and pre‐existing health conditions were not included due to data limitations. The omission of these variables may result in residual confounding and limit the comprehensiveness of the analysis.

Fourth, although multiple count regression models were evaluated, the results may still be sensitive to model specification and underlying assumptions, particularly given the relatively small sample size and the partially constructed nature of the outcome data. Additional modeling approaches and sensitivity analyses could further strengthen the robustness of the findings.

Finally, the use of secondary data sources and the limited geographic and temporal scope may affect the external validity of the results. Therefore, caution should be taken when generalizing these findings beyond the study context.

## 5 Conclusion

Heatstroke represents a significant public health concern, particularly in regions experiencing extreme climatic conditions. The findings of this study revealed that both the maximum temperature and relative humidity are positively associated with heatstroke mortality in Bangladesh during the pre‐monsoon period. The results indicate that rises in these climatic factors are linked with higher rates of heatstroke related deaths. The findings also highlight the importance of both the temperature and humidity in dealing with heat related health risks and in designing effective public health interventions. In Bangladesh, high temperature and humidity frequently coincide. Therefore, the improvement of heat health warning systems and targeted interventions strategies is essential to reduce heat related mortality. However, the findings should be interpreted considering the study's limitations, including the short study period and partial reliance on constructed daily mortality data. Future research using long‐term data and incorporating other environmental and socioeconomic factors is needed to provide in depth understanding of heatstroke mortality.

Overall, the present study provides evidence to support climate‐sensitive public health planning and underscores the need for adaptive strategies to mitigate the health impacts of rising temperatures in Bangladesh and similar settings.

## Author Contributions

**Md Mamun Miah:** conceptualization, methodology, software, data curation, investigation, validation, supervision, resources, project administration, visualization, writing – review and editing, writing – original draft. **Farjana Haque Pingki:** conceptualization, investigation, formal analysis, validation, visualization, writing – review and editing, software, methodology, writing – original draft. **Sarmin Akhter:** data curation, formal analysis, writing – original draft. **Mehedi Hasan:** formal analysis, writing – original draft, writing – review and editing, visualization. **Minhaz Uddin:** data curation, formal analysis, writing – original draft, writing – review and editing. **Rakib Rahman:** writing – original draft, visualization, data curation, formal analysis. **Tanzir Ahamed Raihan:** writing – original draft, formal analysis, visualization. **Md. Shiblur Rahaman:** supervision, writing – review and editing.

## Funding

The authors have nothing to report.

## Ethics Statement

This study utilized publicly available secondary data from the Directorate General of Health Services (DGHS), Bangladesh, and the NASA POWER Data Access Viewer. As no individual‐level or identifiable data were used, ethical approval and informed consent were not required.

## Consent

The authors have nothing to report.

## Conflicts of Interest

The authors declare no conflicts of interest.

## Transparency Statement

The lead author Md. Mamun Miah affirms that this manuscript is an honest, accurate, and transparent account of the study being reported; that no important aspects of the study have been omitted; and that any discrepancies from the study as planned (and, if relevant, registered) have been explained.

## Data Availability

The data sets are publicly available at GitHub repository: https://github.com/STAT-Mamun/Heatstroke-data/blob/main/data.xlsx.
